# Enhanced Anti-Tumoral Activity of Methotrexate-Human Serum Albumin Conjugated Nanoparticles by Targeting with Luteinizing Hormone-Releasing Hormone (LHRH) Peptide

**DOI:** 10.3390/ijms12074591

**Published:** 2011-07-15

**Authors:** Azade Taheri, Rassoul Dinarvand, Fatemeh Atyabi, Fatemeh Ahadi, Farank Salman Nouri, Mohammad Hossein Ghahremani, Seyed Nasser Ostad, Atefeh Taheri Borougeni, Pooria Mansoori

**Affiliations:** 1 Department of Pharmaceutics, Faculty of Pharmacy, Tehran University of Medical Sciences, Tehran P. O. Box 14155-6451, Iran; E-Mails: azadetaheri@yahoo.com (A.T.); ahadi83@yahoo.com (F.A.); fsnoori@gmail.com (F.S.N.); 2 Nanotechnology Research Centre, Faculty of Pharmacy, Tehran University of Medical Sciences, Tehran 14174, Iran; E-Mails: atyabifa@tums.ac.ir (F.A.); mhghahremani@tums.ac.ir (M.H.G.); ostadnas@sina.tums.ac.ir (S.N.O.); 3 Department of Pharmacology, Faculty of Pharmacy, Tehran University of Medical sciences, Tehran 14174, Iran; 4 Department of Oral and Maxillofacial Pathology, Faculty of Dentistry, Tehran University of Medical Sciences, Tehran 14174, Iran; E-Mail: atefetaheriborujeni@yahoo.com; 5 Faculty of Medicine, Ahvaz Jundishapur University of Medical Sciences, Ahvaz 73166, Iran; E-Mail: pooriyamansoori@yahoo.com

**Keywords:** nanoparticles, drug targeting, conjugates, anti-cancer, human serum albumin, LHRH

## Abstract

Active targeting could increase the efficacy of anticancer drugs. Methotrexate-human serum albumin (MTX-HSA) conjugates, functionalized by luteinizing hormone-releasing hormone (LHRH) as targeting moieties, with the aim of specifically targeting the cancer cells, were prepared. Owing to the high expression of LHRH receptors in many cancer cells as compared to normal cells, LHRH was used as the targeting ligand in this study. LHRH was conjugated to MTX-HSA nanoparticles via a cross-linker. Three types of LHRH targeted nanoparticles with a mean particle size between 120–138 nm were prepared. The cytotoxicity of LHRH targeted and non-targeted nanoparticles were determined on the LHRH positive and negative cell lines. The internalization of the targeted and non-targeted nanoparticles in LHRH receptor positive and negative cells was investigated using flow cytometry analysis and fluorescence microscopy. The cytotoxicity of the LHRH targeted nanoparticles on the LHRH receptor positive cells were significantly more than non-targeted nanoparticles. LHRH targeted nanoparticles were also internalized by LHRH receptor positive cells significantly more than non-targeted nanoparticles. There were no significant differences between the uptake of targeted and non-targeted nanoparticles to the LHRH receptor negative cells. The active targeting procedure using LHRH targeted MTX-HSA nanoparticles could increase the anti-tumoral activity of MTX.

## 1. Introduction

Breast cancer is the most common malignancy and the second cause of cancer death in women [1,2]. Chemotherapy is the main method for the treatment of metastatic cancers [3]. However, the systemic toxicity of chemotherapeutic agents restricts cancer chemotherapy [4]. Specific targeted chemotherapy could decrease the toxic side effects on healthy cells and improve the efficacy of chemotherapeutic drugs [5–7]. The peptide receptors expressed mainly on tumor cells can serve as targeting tools for a selective drug delivery to malignant tumors [8]. Luteinizing hormone-releasing hormone (LHRH) is a hormonal decapeptide produced by the hypothalamus, which regulates the pituitary-gonadal axis and thus reproduction. LHRH receptors are overexpressed in breast, ovarian, endometrial and prostate cancer cells. In contrast, LHRH receptors are not expressed detectably in normal organs [9–12]. Therefore, LHRH can be used as a targeting agent to enhance anticancer drug uptake to LHRH receptor positive cancerous cells and reduce the toxic side effects of cytotoxic drugs on normal cells.

Several efforts have been made to use LHRH and its analogs as a targeting agent [13–20]. In Nagy and Schally works [13,14], LHRH and its analogs have been used as carrier vectors to deliver cytotoxic drugs to tumor cells. They developed a class of targeted antitumor agents by linking cytotoxic drugs such as cisplatin and doxorubicin to LHRH or LHRH analogs. All of these LHRH cytotoxic drug conjugates showed LHRH-receptor-mediated *in vitro* and *in vivo* anticancer activity and improved the efficacy of their antiproliferative agent compared to the non-targeted antiproliferative agent [15–17]. The cytotoxic drug-LHRH analogs conjugates showed a wide range of specific binding affinities to LHRH receptors and were also internalized to the tumor cells [18,21]. LHRH could be linked as a targeting moiety to colloidal systems such as dendrimers [19] or nanoparticles [20] with high drug loading capacity. Minko and coworkers developed a LHRH targeted poly amidoamine dendrimerpaclitaxel conjugate and showed that LHRH targeted conjugates could be internalized by cancer cells efficiently and reduce the adverse side effects of chemotherapy [20].

Targeting moiety in the LHRH targeted conjugates enhance cellular uptake and internalization of targeted conjugates compared to non-targeted conjugates via receptor-mediated endocytosis [22–24].

Human serum albumin (HSA) represents a biodegradable drug carrier system with the capacity of delivering a large payload of cytotoxic drug to the tumor site [25,26]. Drugs can be incorporated within the HSA nanoparticles [27], or bound with covalent linkage to HSA [28,29]. The free amino groups of HSA could be used for covalent coupling of targeting moieties to the surface of the HSA nanoparticles [30–32]. Therefore HSA nanoparticles have been proposed as a suitable drug carrier system for targeted drug delivery to specific sites [33].

In our previous study we developed methotrexate-human serum albumin conjugated (MTX-HSA) nanoparticles a delivery system, and showed that they could be used to deliver cytotoxic drug to tumor cells more efficiently when compared with the free cytotoxic drug [28].

This investigation was performed to determine whether covalent attachment of LHRH molecules on the surface of MTX-HSA nanoparticles as a targeting moiety would improve the cytotoxic effect of MTX-HSA nanoparticles on LHRH receptor positive tumor cells. The uptake of LHRH targeted MTX-HSA nanoparticles to LHRH receptor positive cells, T47D cells [34] and LHRH receptor negative cells, SKOV3 cells [19] were investigated *in vitro*.

## 2. Results

### 2.1. Characterization of LHRH Targeted MTX-HSA Nanoparticles

In order to attach LHRH to MTX-HSA nanoparticles, 1-ethyl-3-(3-dimethylaminopropyl) carbodiimide HCl (EDC) cross-linker was used, which could form amide linkage between the amino groups of LHRH molecules and COOH groups on the surface of MTX-HSA nanoparticles.

**Figure 1:**
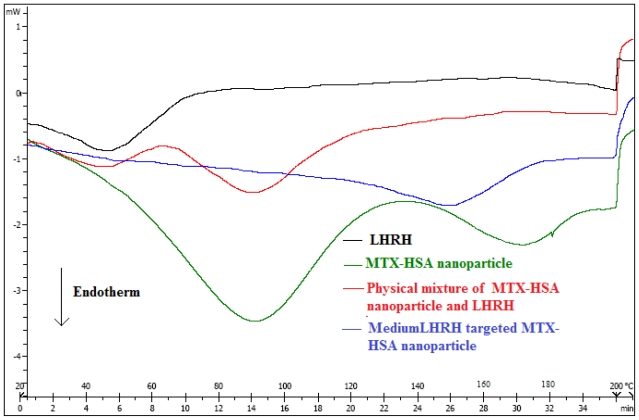
Differential scanning calorimetric (DSC) thermograms of luteinizing hormone-releasing hormone (LHRH), methotrexate-human serum albumin (MTX-HSA) nanoparticles, physical mixture of LHRH and MTX-HSA nanoparticles, and medium LHRH targeted MTX-HSA nanoparticles.

Figure 1 shows the differential scanning calorimetric (DSC) thermograms of LHRH, MTX-HSA nanoparticles, physical mixture of LHRH and MTX-HSA nanoparticles, and medium LHRH targeted MTX-HSA nanoparticles. DSC technique could support the conjugation of LHRH to MTX-HSA nanoparticles [35–37]. The pure LHRH, MTX-HSA nanoparticles and physical mixture of LHRH and MTX-HSA nanoparticles were analyzed as control samples using DSC. The endothermic peaks of physical mixture of LHRH and MTX-HSA nanoparticles (48 °C and 92 °C) are similar to endothermic peaks of pure LHRH (50 °C) and MTX-HSA nanoparticles (91 °C), whereas the LHRH targeted MTX-HSA nanoparticles showed an endothermic peak at 149 °C.

LHRH targeted MTX-HSA nanoparticles were prepared at three different LHRH/HSA molarity ratio: 5.8 ± 0.12 which nominated as low LHRH targeted MTX-HSA nanoparticles, 17.3 ± 0.23 as medium LHRH targeted MTX-HSA nanoparticles and 29.1 ± 0.2 as high LHRH targeted MTX-HSA nanoparticles. The results of the physico-chemical characterization of LHRH targeted MTX-HSA nanoparticles are presented in Table 1.

The non-targeted MTX-HSA nanoparticles (MTX/HSA molar ratio = 8) were characterized by a size of 111.7 ± 4.6 nm whereas the LHRH targeted MTX-HSA nanoparticles showed a higher size in the range of 120.5 ± 2.7 to 138.56 ± 3.2 nm according to the number of attached LHRH molecules. All types of nanoparticles showed a monodispersed size distribution. The zeta potential of non-targeted MTX-HSA nanoparticles was −12.1 ± 0.5 mV whereas the zeta potential of the LHRH targeted MTX-HSA nanoparticles was about −10.45 ± 1.23 mV.

### 2.2. Transmission Electron Microscopy (TEM)

**Figure 2:**
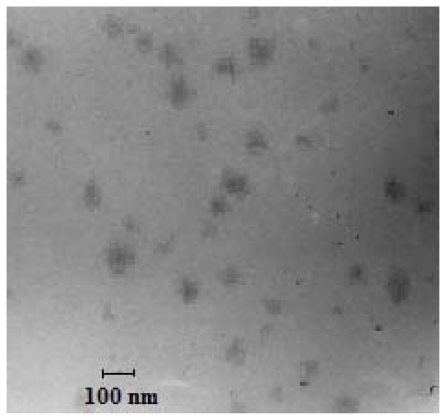
TEM image of medium LHRH targeted MTX-HSA nanoparticles.

Figure 2 shows the TEM graph of low LHRH targeted MTX-HSA nanoparticles. The nanoparticles were almost spherical with diameters less than 100 nm Lower than the size obtained by laser light scattering measurement. The dehydration of LHRH targeted MTX-HSA nanoparticles during TEM experiment may be the reason for the smaller size of nanoparticles obtained by TEM method compared to laser light scattering [37].

### 2.3. Stability of LHRH Targeted MTX-HSA Nanoparticles

**Figure 3:**
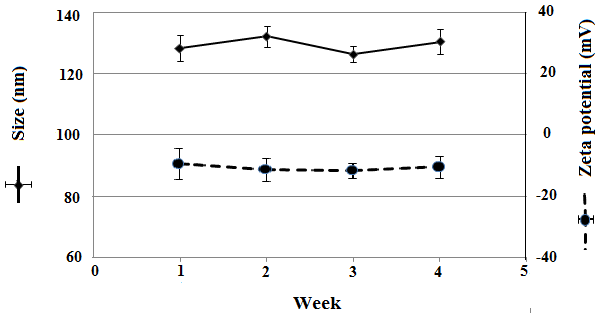
Storage stability of medium LHRH targeted MTX-HSA nanoparticle. Size and zeta potential of nanoparticles were recorded for 4 weeks.

The release of MTX and LHRH from LHRH targeted MTX-HSA nanoparticles were calculated in aqueous buffered solution at 4 °C for 4 weeks. The stability of the LHRH targeted MTX-HSA nanoparticles under mentioned condition was high and after 4 weeks at least 98.2 ± 1.1% of methotrexate and 99.1 ± 2.1% of LHRH were still attached to the HSA for high LHRH targeted MTX-HSA nanoparticles. Same results were obtained for the low and medium LHRH targeted MTX-HSA nanoparticles. Moreover, the size and zeta potential of LHRH targeted MTX-HSA nanoparticles did not change significantly at the storage period (Figure 3). Therefore, the LHRH targeted MTX-HSA nanoparticles were considered stable over the observed time.

### 2.4. *In Vitro* Cytotoxicity

**Figure 4:**
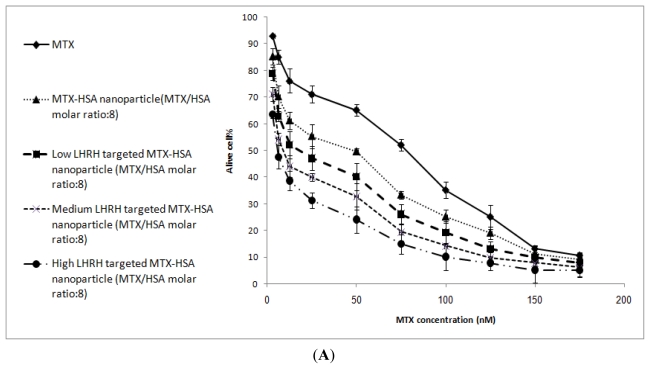

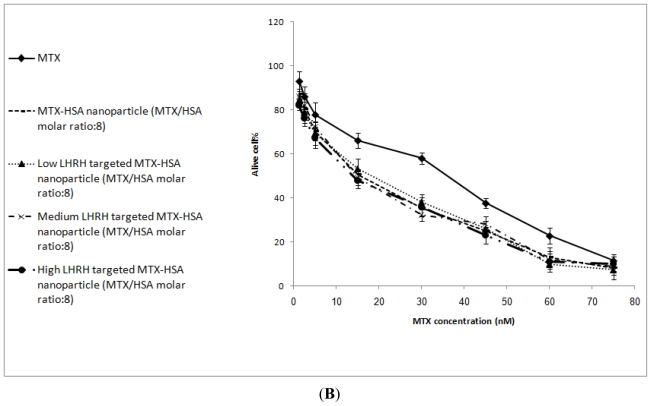
*In vitro* cytotoxicity of MTX, MTX-HSA nanoparticles (MTX/HSA molar ratio: 8) and low, medium and high LHRH targeted MTX-HSA nanoparticles (MTX/HSA molar ratio: 8) on the LHRH receptor positive T47D cells (**A**) and LHRH receptor negative SKOV3 cells (**B**) for 24.

According to the results of cytotoxicity test, free LHRH had no cytotoxic effect on the cells. As shown in Figure 4A, LHRH targeted MTX-HSA nanoparticles were significantly more cytotoxic than non-targeted MTX-HSA nanoparticles on the LHRH receptor positive T47D cells.

The average IC50 values (the dose which produces 50% inhibition of growth) for free MTX, MTX-HSA nanoparticles, low, medium and high LHRH targeted MTX-HSA nanoparticles on the LHRH receptor positive T47D cells were 78.23 ± 3.12, 49.2 ± 2.12, 19.3 ± 1.98, 9.12 ± 1.36, 5.82 ± 1.08 nM respectively (Table 2).

It was obvious that the cytotoxicity of LHRH targeted MTX-HSA nanoparticles were significantly greater than non-targeted MTX-HSA nanoparticles on the LHRH receptor positive T47D cells after 24 h. In addition the cytotoxicity of LHRH targeted MTX-HSA nanoparticles was increased proportionate to the number of attached LHRH molecules on the MTX-HSA nanoparticles on the LHRH receptor positive T47D cells after 24 h. Moreover significant difference between the cytotoxicity of the LHRH targeted MTX-HSA nanoparticles and non-targeted MTX-HSA nanoparticles were observed at lower concentration.

Figure 4B show that there were no significant differences between the cytotoxicity of LHRH targeted MTX-HSA nanoparticles and non-targeted MTX-HSA nanoparticles on the LHRH receptor negative SKOV3 cells after 24 h. The IC50 values of LHRH targeted MTX-HSA nanoparticles and non-targeted MTX-HSA nanoparticles were almost similar on the LHRH receptor negative SKOV3 cells after 24 h (Table 2).

### 2.5. Cellular Uptake of LHRH Targeted MTX-HSA Nanoparticles

The cellular uptake of the MTX-HSA nanoparticles and LHRH targeted MTX-HSA nanoparticles were evaluated using flow cytometry analysis. LHRH receptor positive T47D cells and LHRH receptor negative SKOV3 cells were incubated with LHRH targeted MTX-HSA nanoparticles and non-targeted MTX-HSA nanoparticles.

**Figure 5:**
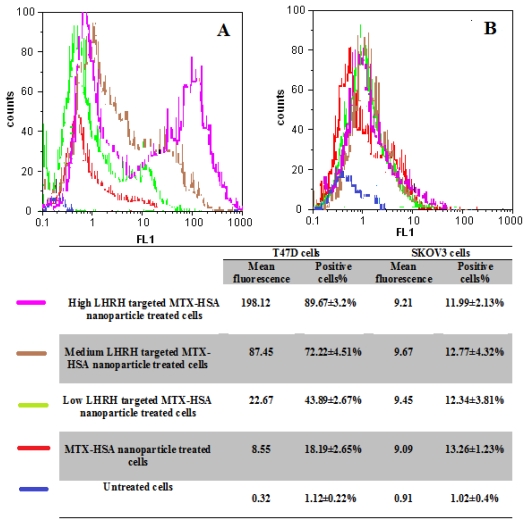
The amount of fluorescence exhibited from LHRH receptor positive T47D cells (**A**) and LHRH receptor negative SKOV3 cells (**B**) SKOV3 cells treated with low, medium and high LHRH targeted MTX-HSA nanoparticles and non-targeted MTX-HSA nanoparticles at 37 °C, for 5 h.

As shown in Figure 5A, there was a specific targeting with LHRH targeted MTX-HSA nanoparticles for LHRH receptor positive T47D cells. The result showed 43.89 ± 2.67% fluorescence positive cells for the low LHRH targeted MTX-HSA nanoparticle treated cells, 72.22 ± 4.51% fluorescence positive cells for the medium LHRH targeted MTX-HSA nanoparticle treated cells and 89.67 ± 3.2% fluorescence positive cells for the high LHRH targeted MTX-HSA nanoparticle treated cells. The cellular uptake of LHRH targeted MTX-HSA nanoparticles by the LHRH receptor positive T47D cells increased by addition of the number of attached LHRH on the surface of MTX-HSA nanoparticles (Figure 5A). However, non-targeted MTX-HSA nanoparticles showed lower cellular uptake (18.19 ± 2.65% fluorescence positive cells).

When the LHRH receptor negative SKOV3 cells were incubated with the LHRH targeted MTX-HSA nanoparticle and non-targeted MTX-HSA nanoparticles, there was no significant difference between the fluorescence positive cells for the LHRH targeted MTX-HSA nanoparticle and non-targeted MTX-HSA nanoparticles treated SKOV3 cells (Figure 5B).

To confirm LHRH receptor-mediated uptake of the LHRH targeted MTX-HSA nanoparticle, the uptake of different types of the LHRH targeted MTX-HSA nanoparticle was evaluated by fluorescence microscopy in LHRH receptor positive T47D cells and LHRH receptor negative SKOV3 cells. The cells were incubated with non-targeted MTX-HSA nanoparticles and low, medium and high LHRH targeted MTX-HSA nanoparticles (1 mg/mL) at 37 °C for 4 h.

**Figure 6:**
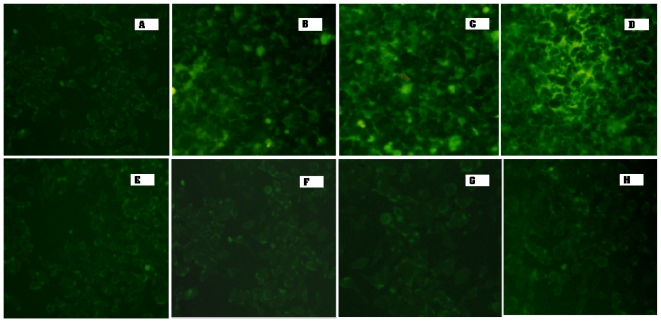
*In vitro* evaluation of targeting the LHRH targeted MTX-HSA nanoparticles to LHRH receptor positive T47D cells determined by fluorescence microscopy. The LHRH receptor positive T47D cells were incubated with non-targeted MTX-HSA nanoparticles (**A**), low LHRH targeted MTX-HSA nanoparticles (**B**), medium LHRH targeted MTX-HSA nanoparticles (**C**), high LHRH targeted MTX-HSA nanoparticles (**D**), for 4 h at 37 °C. Higher fluorescence from the LHRH targeted MTX-HSA nanoparticles compared to fluorescence from the non-targeted MTX-HSA nanoparticles could indicate the higher uptake of LHRH targeted MTX-HSA nanoparticles by T47D cells compared to non-targeted MTX-HSA nanoparticles. The amount of fluorescence exhibited from LHRH targeted MTX-HSA nanoparticles treated T47D cells is proportionate to the number of attached LHRH molecules on the surface of MTX-HSA nanoparticles (B, C and D). The LHRH receptor negative SKOV3 cells were incubated with non-targeted MTX-HSA nanoparticles (**E**), low LHRH targeted MTX-HSA nanoparticles (**F**), medium LHRH targeted MTX-HSA nanoparticles (**G**), high LHRH targeted MTX-HSA nanoparticles (**H**) for 4 h at 37 °C. There are no significant differences between the amounts of fluorescence exhibited from SKOV3 cells treated with non-targeted and targeted MTX-HSA nanoparticles (E, F, G and H).

In the LHRH receptor positive T47D cells (Figure 6A–D), incubation with LHRH targeted MTX-HSA nanoparticles (Figure 6B–D) showed more cellular uptake than non-targeted MTX-HSA nanoparticles (Figure 6A). The fluorescence exhibited from LHRH targeted MTX-HSA nanoparticles treated cells (37 °C for 4 h) was proportionate to the number of LHRH molecules conjugated on the surface of MTX-HSA nanoparticles. The high LHRH targeted MTX-HSA nanoparticles treated cells exhibited more fluorescence than low and medium LHRH targeted MTX-HSA nanoparticles treated cells. In contrast, in the case of LHRH receptor negative SKOV3 cells, the conjugation of LHRH on the surface of MTX-HSA nanoparticles did not improved the uptake of LHRH targeted MTX-HSA nanoparticles (Figure 6F–H) compared to non-targeted MTX-HSA nanoparticles (Figure 6E).

## 3. Discussion

The adverse side effect of cytotoxic drugs could limit the efficacy of tumor chemotherapy [4]. The specific targeted drug delivery systems could decrease the cytotoxicity of anti-cancer drugs in healthy organs [8,12,38].

LHRH receptors are overexpressed in several types of cancer cells but the expression of these receptors on the healthy cells is rare [10,39]. Moreover, LHRH receptors could induce the internalization of the LHRH targeted drug delivery systems by the cancer cells specifically [40]. Thus, LHRH peptide could be used as a suitable targeting moiety for specific drug delivery to LHRH receptor overexpressing tumors [10,11,40]. In the Xu *et al.* study [41], LHRH functionalized FePt nanoparticles were prepared. The cytotoxicity of LHRH functionalized FePt nanoparticles were evaluated on the LHRH receptor positive human ovarian cancer cell line (A2780). The results showed that LHRH functionalized FePt nanoparticles had more cytotoxic effect on the LHRH receptor positive tumor cells in comparison with non-functionalized FePt nanoparticles. In another study [40], Dharap *et al.* synthesized poly ethyleneglycol (PEG)-camptothecin (CPT) and LHRH-PEG-CPT conjugates. The cytotoxicity of conjugates was studied on the A2780 human ovarian cancer cells. The results of cytotoxicity test showed that the LHRH targeted conjugates were more cytotoxic on the LHRH receptor positive A2780 cells than non-targeted conjugates [40]. Moreover, Bajo *et al*. showed that LHRH peptide-cytotoxic drug conjugates could improve the cytotoxic effect of drugs on the drug resistant tumor cells [42]. In their study doxorubicin (DOX) was linked to D-Lys6 LHRH (AN-152). The cytotoxic effect of this conjugate was evaluated on the LHRH receptor positive and DOX-resistant human mammary carcinoma line MX-1, into nude mice bearing MX-1 tumors. The results showed that, the final tumor volumes of the AN-152 treated mouse were significantly smaller than the final tumor volumes of the free DOX treated mouse. It may be concluded that the AN-152 cytotoxic conjugate could target the LHRH receptor positive and DOX resistant tumor cells and improve DOX cytotoxicity.

HSA have been used as suitable delivery carriers for anti-cancer drugs in many studies [25,43,44], and its NH2 groups have been used as linking point for conjugation of cytotoxic drugs [29,45,46]. To achieve a specific targeted drug delivery system, the surface of HSA nanoparticles could be decorated by targeting agents covalently [37,43,44,47].

In our previous work, we developed MTX conjugated HSA nanoparticles [28]. In this study, LHRH targeted MTX-HSA nanoparticles were prepared via a carbodiimide mediated coupling of NH2 groups of LHRH to COOH groups of the MTX-HSA nanoparticles. The covalent attachment of LHRH molecules to MTX-HSA nanoparticles was proved by the results of the DSC analysis. The changes in the thermal behavior of the LHRH targeted MTX-HSA nanoparticles in comparison with the pure LHRH and MTX-HSA nanoparticles could support the covalent attachment of LHRH molecules to the MTX-HSA nanoparticles. Three types of LHRH targeted MTX-HSA nanoparticles different in the LHRH/HSA molar ratios were prepared. After LHRH conjugation, the size of MTX-HSA nanoparticles were increased proportionate to the amount of attached LHRH molecules and the zeta potential of nanoparticles were increased slightly (−10.45 ± 1.23 mV). The slightly increase in the zeta potential of the LHRH targeted MTX-HSA nanoparticles may be attributed to the decrease of the free COOH groups on the MTX-HSA nanoparticles after conjugation of LHRH peptide to the COOH groups of the MTX-HSA nanoparticles. TEM images of LHRH targeted MTX-HSA nanoparticles showed the narrow size distribution of these nanoparticles. The stability of the LHRH targeted MTX-HSA nanoparticles under examined condition (4 °C for four weeks) were high. The results of the stability test showed that LHRH targeted MTX-HSA nanoparticles could remain unchanged at the storage period. A LHRH receptor positive cell line (T47D) and a LHRH receptor negative cell line (SKOV3) were used to evaluate the cytotoxic activity of the LHRH targeted MTX-HSA nanoparticles and non-targeted MTX-HSA nanoparticles. The cytotoxicity results showed that the LHRH targeting procedure could increase significantly the anti-tumoral activity of LHRH targeted MTX-HSA nanoparticles in comparison with non-targeted MTX-HSA nanoparticles and free MTX on the LHRH receptor positive T47D cells. The high, medium and low LHRH targeted MTX-HSA nanoparticles had 8.45 ± 0.02, 5.39 ± 0.02 and 2.54 ± 0.04 times lower IC50 value than non-targeted MTX-HSA nanoparticles after 24 h on the LHRH receptor positive T47D cells. No significant effect was seen for the non-targeted nanoparticles on the LHRH receptor negative SKOV3 cells. LHRH targeted MTX-HSA nanoparticles could specifically bind to the LHRH receptor and could be internalized by receptor-mediated endocytosis [48]. Endocytosis of the LHRH targeted MTX-HSA nanoparticles into the LHRH receptor positive cells could increase the amount of delivered cytotoxic drugs to the targeted cells. The flow cytometry results showed that the LHRH targeted MTX-HSA nanoparticles possessed high internalization activity by LHRH receptor positive cells. Also the internalization efficiencies of the LHRH receptor positive T47D cells for the LHRH targeted MTX-HSA nanoparticles were proved by the results of fluorescence microscopy. The amount of the exhibited FITC fluorescence from the FITC labeled LHRH targeted MTX-HSA nanoparticles treated T47D cells were significantly more than the FITC labeled non-targeted MTX-HSA nanoparticles. Moreover, the amount of the exhibited FITC fluorescence from the FITC labeled LHRH targeted MTX-HSA nanoparticles treated T47D cells increased proportionate to the number of attached LHRH molecules on the MTX-HSA nanoparticles. However, there were no significant difference between the amounts of the exhibited FITC fluorescence from the FITC labeled LHRH targeted MTX-HSA nanoparticles treated SKOV3 cells and FITC labeled LHRH non targeted MTX-HSA nanoparticles treated SKOV3 cells.

## 4. Experimental Section

### 4.1. Materials

LHRH, Human serum albumins (HSA), 1-ethyl-3-(3-dimethylaminopropyl) carbodiimide HCl (EDC), fluorescein isothiocyanate (FITC), *N*-hydroxy succinimide were all purchased from Sigma (Steinheim, Germany). Methotrexate USP was kindly donated by Cipla Pharmaceutical Co, India. Total protein kit (Micro Lowry) was from Sigma (Saint Louis, USA). T47D, a human breast cancer cell line and SKOV3, a human ovarian cancer cell line, were obtained from American Type Culture. PMRI-1640 modified medium and penicillin/streptomycin solution was obtained from Gibco Invitrogen (Calsbad, CA, USA). MTT was obtained from Sigma (St. Louis, USA). All other reagents were of analytical grade. Deionized water was used throughout the experiment.

### 4.2. Preparation of LHRH Targeted MTX-HSA Nanoparticles

Preparation of MTX-HSA conjugates: MTX (20 mg) with EDC (15 mg) was dissolved in dimethylsulfoxide (DMSO) (1.25 mL). The solution was placed in water bath at 50 °C for 15 min. Then the solution was cooled to room temperature and was added to HSA solution (250 mg HSA in 2 mL phosphate buffer solution (PBS) pH 7.4) at the rate of 1 mL/min while stirring at 600 rpm. After reaction time (20 min), unreacted MTX, EDC, DMSO and unreacted HSA were removed using Amicon Ultra-4 Centrifugal Filter Devices with cutoff 100 kDa (Millipore, USA).

Preparation of MTX-HSA nanoparticles: The MTX-HSA conjugates so formed were cross-linked by adding 5 mg EDC under constant stirring (600 rpm) at room temperature for 4 h to produce MTX-HSA nanoparticles. Dialysis against PBS was performed (cellulose membrane, cutoff 12000 Da from Merck, Germany) to remove small molecules such as unreacted EDC, DMSO, *etc.* Nanoparticles were then lyophilized at −40 °C for 48 h (Lyotrap Plus, LTE, Scientific ltd, Oldham, UK) [28]. We prepared LHRH targeted MTX-HSA nanoparticles by MTX-HSA nanoparticles with different MTX/HSA molar ratios. In this study, we used MTX-HSA nanoparticles with MTX/HSA molar ratio: 8 for targeting experiments [28]. 2, 5 and 10 mg of LHRH were added to MTX-HSA nanoparticles (MTX/HSA molar ratio: 8) (25 mg in 1 mL water) and mixed. 250 μL of a freshly prepared EDC solution (10 mg in 1 mL of water) was added to LHRH and MTX-HSA nanoparticles mixture, and the solution was vortexed for 10 min. The pH of the mixture was kept close to 6 with NaOH. Then the mixture was left on a rolling mixer in a dark, cold room at 4 °C for 15 h. After the reaction time, unreacted EDC and LHRH were removed using Amicon^®^ Ultra-4 Centrifugal Filter Devices with cutoff 30 kDa (Millipore, USA). The amounts of EDC used for conjugation of MTX or LHRH to HSA were optimized to prevent any aggregation. Also gel filtration using Sephadex G**-**25 column (Little Chalfont, Buckinghamshire, UK) was used to separate any possible aggregate. When we used Sephadex G**-**25 column for aggregate separation, unreacted agents (EDC or LHRH) from LHRH targeted MTX-HSA nanoparticles (if remained). To ensure the separation of the soluble LHRH-HSA-MTX and MTX-HSA (if present), Amicon Ultra-4 Centrifugal Filter Devices with cutoff 100 kDa (Millipore, USA) were used with the combination of gel chromatography using sephacryl® 300 HR. LHRH targeted MTX-HSA nanoparticles were then lyophilized at −40 °C for 48 h (Lyotrap Plus, LTE, Scientific Ltd., Oldham, UK).

### 4.3. Determination of LHRH on the Surface of LHRH Conjugated MTX-HSA Nanoparticles

Total protein kit (Micro Lowry) was used for determination of the amount of LHRH coupled to MTX-HSA nanoparticles. The total amount of LHRH bound to the MTX-HSA nanoparticles was calculated as the difference between the total amount of LHRH used for conjugation and the amount of unreacted LHRH determined after the filtration step described above.

### 4.4. Characterization of Nanoparticles

#### 4.4.1. Particle Size and Zeta Potential Measurement

Lyophilized LHRH targeted MTX-HSA nanoparticles were dispersed in PBS at the concentration of 1 mg/mL. The particle size and zeta potential of LHRH conjugated MTX-HSA nanoparticles were determined by Zetasizer Nano ZS (Malvern Instruments, UK).

#### 4.4.2. Transmission Electron Microscopy (TEM) Observation

Morphological observation of Lyophilized LHRH targeted MTX-HSA nanoparticles were performed using a transmission electron microscope (TEM) (Zeiss EM 900, Jena, Germany) at a voltage of 8 kV.

#### 4.4.3. Stability of LHRH Targeted MTX-HSA Nanoparticles

The LHRH targeted MTX-HSA nanoparticles samples were stored in PBS pH 7.4 (25 mg in 4 cc) at 4 °C for a period of four weeks. Every week an aliquot (100 μL) of the nanoparticle suspension was diluted to 2 mL using PBS pH 7.2 centrifuged using Amicon^®^ Ultra-4 Centrifugal Filter Devices (cutoff: 30 kDa) (Millipore, USA) and the filtrate was analyzed for unbound LHRH using the total protein kit (Micro Lowry).The amount of free methotrexate were determined using high performance liquid chromatography (HPLC) on a Teknokroma C18, 4.6 × 250 mm column at 305 nm. A mixture of phosphate buffer pH 7.2: acetonitrile (88:12 v/v) at a flow rate of 1.20 mL/min was used as the mobile phase [28]. Particle size and zetapotential of nanoparticles were measured additionally.

#### 4.4.4. Differential Scanning Calorimetric (DSC) Analysis

The conjugation of LHRH molecules to MTX-HSA nanoparticles was studied by differential scanning calorimetric (DSC) analysis (DSC-60, Shimadzu, Japan). Approximately 5 mg of LHRH, MTX-HSA nanoparticles, physical mixture of LHRH and MTX-HSA nanoparticles, and LHRH targeted MTX-HSA nanoparticles were placed in aluminum pans. The samples were heated over a range of 20 °C to 200 °C at a rate of 10 °C per min and the thermal behavior of samples were recorded.

### 4.5. Cell Culture

Human breast cancer cells (T47D) and ovary cancer cells (SKOV3) were cultured in T-75 flasks at 37 °C in a humidified incubator containing 5% CO_2_ using RPMI-1640 medium supplemented with 10% heat-inactivated fetal bovine serum and 1% penicillin–streptomycin calf serum. Cells were maintained in an exponential growth phase by periodic sub-cultivation. The medium was replenished every day.

### 4.6. *In Vitro* Cytotoxicity Assay

*In vitro* cytotoxicity of the MTX-HSA nanoparticles and LHRH targeted MTX-HAS nanoparticles were tested on T47D and SKOV-3 cells using the MTT test. T47D and SKOV-3 cells were seeded in 96-well plates (Costar, IL, USA) at a density of 20,000 cells per well and incubated for 24 h. For free drug, a stock solution of methotrexate was prepared in NaOH 0.1 N (5mg/mL), and suitable concentrations were obtained by dilution of the initial stock solution in culture medium. The suspensions of MTX-HSA nanoparticles and LHRH targeted MTX-HSA nanoparticles were diluted in culture medium at equivalent methotrexate concentrations ranging from 3.12–200 nM. The cell culture medium was then removed and replaced by fresh medium containing various amounts of methotrexate, MTX-HSA nanoparticles, LHRH targeted MTX-HSA nanoparticles, and free LHRH. The cells were incubated 24 h at 37 °C. After mentioned time, 20 μL of MTT (5 mg/mL in PBS) was added to each well. After 3–4 h the culture medium containing MTT solution was removed. One hundred μL of DMSO was added to each well to dissolve the formazan crystals and then plates were analyzed using a microplate reader (anthos 2020, Austria) at 570 nm. Cell viability was calculated by comparing the samples to cells incubated with normal culture medium as 100% survival rate.

### 4.7. Flow Cytometry Experiment

Cellular targeting and uptake of non-targeted and LHRH targeted nanoparticles were investigated using flow cytometry. Flow cytometry analysis was performed on a flow cytometer (Partec PAS, Germany). LHRH targeted MTX-HSA nanoparticles and non-targeted MTX-HSA nanoparticles were labeled by FITC. Briefly, 1 mg of nanoparticles was resuspended in deionized water. 125 μL of PBS pH 7.2 and 125 μL of carbonate buffer pH 9 and 62 μL of FTIC solution (2 mg in 1 cc DMSO) were added to nanoparticle solution. Unbound FITC molecules were removed. FITC labeled LHRH targeted MTX-HSA nanoparticles and FITC-labeled MTX-HSA nanoparticles were suspended in RPMI-1640 medium and added to monolayers of SKOV3 cells (300,000) and T47D cells (300,000) grown in 24-well plastic dishes (Costar, IL, USA). The cells were then incubated with FITC-labeled non-targeted and LHRH targeted MTX-HSA nanoparticles in a concentration of 1 mg/ml at 37 °C for 5 h. The cells were then thoroughly washed three times with PBS (pH 7.4), trypsinized and resuspended in cold PBS (pH 7.4). As a control, cells were grown separately in the absence of nanoparticles. Then 10,000 cells were analyzed using the flow cytometer. The samples were analyzed with Flomax^®^ software (Partec, Germany).

### 4.8. Fluorescence Microscopy

To study cellular uptake of FITC labeled MTX-HSA nanoparticles and LHRH targeted MTX-HSA nanoparticles by the fluorescence microscopy, the T47D and SKOV3 cells were cultured at a density of 300,000 cells per well in 6-well plastic dishes (Costar, IL, USA) for 24 h. The FITC labeled MTX-HSA nanoparticles and LHRH targeted MTX-HSA nanoparticles were added to the cell culture media at a concentration of 1 mg/mL. After 4 h of incubation of the FITC-labeled MTX-HSA nanoparticles and LHRH targeted MTX-HSA nanoparticles at 37 °C, the cells were washed three times with PBS. The specific uptake of nanoparticles by the cells was evaluated by fluorescence microscopy (Olympus IX 71, Japan).

## 3. Conclusions

In this study, a simple and rapid method was developed for the preparation of a LHRH targeted MTX-HSA conjugated nanoparticles with particle size of 120 nm to 138 nm and a slightly negative zeta potential. The active targeting procedure using LHRH targeted MTX-HSA nanoparticles could increase significantly the anti-tumoral activity of MTX at low concentrations in comparison with non-targeted MTX-HSA nanoparticles and free MTX. The results of the present study suggest that the LHRH targeted MTX-HSA nanoparticles could be more effective than non-targeted MTX-HSA nanoparticles against the LHRH receptor positive tumor cells.

## Figures and Tables

**Table 1:** Physico-chemical characteristics of MTX-HSA nanoparticles (MTX/HSA molar ratio = 8) and LHRH targeted MTX-HSA nanoparticles (mean ± SD; *n* = 3).

	Non Targeted MTX-HSA Nanoparticle	Low LHRH Targeted MTX-HSA Nanoparticles	Medium LHRH Targeted MTX-HSA Nanoparticles	High LHRH Targeted MTX-HSA Nanoparticles
**Particle Size (nm)**	111.7 ± 4.6	120.5 ± 2.7	128.45 ± 4.4	138.56 ± 3.2
**Poly Dispersity**	0.10 ± 0.01	0.12 ± 0.09	0.2 ± 0.04	0.14 ± 0.05
**Zeta Potential (mV)**	−12.1 ± 0.5	−10.45 ± 1.23	−10.1 ± 1.1	−10.04 ± 0.65
**LHRH/HSA Molar Ratio**	------	5.8 ± 0.12	17.3 ± 0.23	29.1 ± 0.2

**Table 2:** IC_50_ values (the dose (nM) which produces 50% inhibition of growth) of free MTX, MTX-HSA nanoparticles, low, medium and high LHRH targeted MTX-HSA nanoparticles on the LHRH receptor positive T47D cells and LHRH receptor negative SKOV3 cells after 24 h.

	Free MTX	Non Targeted MTX-HSA Nanoparticles	Low LHRH Targeted MTX-HSA Nanoparticles	Medium LHRH Targeted MTX-HSA Nanoparticles	High LHRH Targeted MTX-HSA Nanoparticle
**T47D cells**	78.23 ± 3.12	49.2 ± 2.12	19.3 ± 1.98	9.12 ± 1.36	5.82 ± 1.08
**SKOV3 cells**	35.89 ± 1.46	15.12 ± 2.19	17.64 ± 2.23	14.89 ± 2.78	14.53 ± 1.46
